# A novel method and classification criteria for analyzing urine turbidity and its relationship with urine dry chemical parameters

**DOI:** 10.1371/journal.pone.0323351

**Published:** 2025-05-07

**Authors:** Jingnan Liu, Yaxing Cheng, Zijuan Zhang, Lanxin Zhu, Liping Pan, Hang Zhou, Huihui Zhao, Xiaoqiao Ren

**Affiliations:** 1 School of Traditional Chinese Medicine, Beijing University of Chinese Medicine, Beijing, China; 2 Institute of Ethnic Medicine and Pharmacy, Beijing University of Chinese Medicine, Beijing, China; UniCamillus: Saint Camillus International University of Health and Medical Sciences, ITALY

## Abstract

**Background:**

Urine turbidity is a significant diagnostic marker for early screening of urinary tract infections, kidney stones, and other related conditions. However, current methods for analyzing urine turbidity often rely on subjective assessments. This study aims to investigate the relationship between urine turbidity and the urine color values measured by spectrophotometer, providing an objective quantification method for both urine turbidity and urine color, while also exploring the underlying causes of urine turbidity.

**Methods:**

A cross-sectional study was conducted among newly enrolled university students undergoing physical examination in Beijing. Basic demographic information and morning urine samples were collected. Urine turbidity was assessed using human visual evaluation and a urine chemical analyzer, while urine color CIE L*a*b* (International Commission on illumination) was measured using a spectrophotometer. Routine urine chemical examination was also performed Correlations among urine turbidity, urine color, and urine dry chemical parameters were analyzed.

**Results:**

A total of 1220 participants (68.7% female, mean age: 23.66 years) were included in the study. Spearman correlation analysis showed that urine turbidity was significantly negatively correlated with L* (lightness) and significantly positively correlated with a* (redness) and b* (yellowness). Regression analysis identified L* as the most affected parameter by urine turbidity (standardized coefficient β=-1.030, *p* < 0.05). Receiver operating characteristic (ROC) analysis showed that L* was highly effective in distinguishing different urine turbidity levels, with L* < 89.165 achieving excellent sensitivity and specificity (AUC = 0.984) and 96% accuracy in identifying turbid urine. In addition, urine turbidity was positively correlated with urine specific gravity, protein, and urine color (*p* < 0.05), while its relationship with pH was nonlinear. These findings suggest that multiple factors collectively influence urine turbidity.

**Conclusion:**

This study provides a novel and objective approach for assessing urine turbidity, advancing the modernization of urine diagnostic practices in traditional medicine.

## Introduction

Urinalysis, encompassing physical, chemical, and microscopic examinations, is a widely used diagnostic test in both modern and traditional medicine [1,2]. In traditional practices such as those of ancient Egypt, Tibetan medicine, and traditional Chinese medicine (TCM), macroscopic parameters like urine color (Ucol) and turbidity are considered diagnostically significant. Changes in these characteristics are believed to reflect the location, nature, and prognosis of various diseases [3]. For instance, in TCM, dark and turbid urine is often interpreted as a sign of excess heat within the body.

Urine turbidity (Uturb) has significant clinical relevance in modern medicine, often serving as an indicator of conditions such as lipiduria, phosphaturia, pyuria, and chyluria. Research has shown that Uturb can aid in diagnosing urinary tract infections (UTIs), urinary stones, et al. [4–12]. For example, a study reported that 90% of 200 adult patients with acute community-acquired UTIs exhibited cloudy urine [13]. In addition urine clarity, when evaluated alongside proteinuria and hematuria, improves diagnostic accuracy for schistosomiasis, especially in low-resource settings [14]. Urine color also correlates with hydration status [15–17] and may reflect overall physical health in healthy adults [18]. Together, Uturb and Ucol play important roles in diagnosis and treatment. Beyond detecting macroscopic changes in Uturb, understanding the underlying causes of these changes is crucial. Thus, exploring the relationship between Uturb and urine parameters is necessary to enhance diagnostic and therapeutic strategies.

Methods for analyzing Uturb primarily include human visual examination and instrumental measurements. Most studies rely on subjective visual evaluation, in which turbidity is assessed based on the legibility of text viewed through a urine container [8–10,19–21]. While convenient, this approach lacks objective quantification and standardization. Instrumental methods, on the other hand, measure turbidity using optical properties, relying on light scattering caused by suspended particles in the urine [22]. Urine analyzers, such as the ARKRAY AX-4030, Shenzhen Mindray UA-5800, and Sysmex UC-3500, classify turbidity into levels (e.g., clear, turbid 1 + , turbid 2+). However, these devices provide qualitative rather than quantitative data and employ varying principles and classification methods. Additionally, they are not widely adopted in China [23,24]. Spectrophotometry offers another approach for measuring Uturb. For example, Livsey S.A. used a double-beam turbidimeter to evaluate Uturb, demonstrating that its effectiveness in detecting infections depends on the turbidity threshold [25]. Similarly, Kovacevic L. et al. employed a dual-beam spectrophotometer to assess turbidity in crystalline solutions, yielding results comparable to those obtained with optical microscopy [26,27]. These findings highlight the objectivity and feasibility of spectrophotometric Uturb measurements, though a universally accepted standard has yet to be established.

Building on this, our study aims to investigate the relationship between Uturb and CIE L*a*b* values measured by spectrophotometry. The CIE L*a*b* color space, recommended by the International Commission on Illumination (CIE), is a three-dimensional model for objectively quantifying color. In this model, L* represents luminance, with higher values indicating greater brightness. The a* axis spans from green (negative values) to red (positive values), while the b* axis ranges from blue (negative values) to yellow (positive values).

To our knowledge, this approach has not been applied to Uturb determination. In this study, we explored the correlations between CIE L*a*b* values and Uturb to establish a quantitative method. Using a large sample size, we identified a cutoff value for Uturb classification and analyzed its relationship with urine dry chemical parameters to determine the factors influencing Uturb.

## Materials and methods

This study adheres to the guidelines outlined in the Strengthening the Reporting of Observational Studies in Epidemiology (STROBE) Statement.

### Study design

This cross-sectional study collected urine samples and basic demographic information during physical examinations to explore the use of spectrophotometry for Uturb analysis. It assessed the relationships between Uturb, Ucol, and other urine parameters.

### Participants

This cross-sectional study recruited newly enrolled students who underwent physical examinations at the Third Affiliated Hospital of Beijing University of Traditional Chinese Medicine from September 2 to September 19, 2021. Morning urine samples were collected during the physical examination, and diet and vitamin intake were not controlled. Data collected included gender, age, and morning urine samples.

The inclusion criteria were as follows: 1) Participation in the physical examination, 2) Female participants who were not menstruating at the time and 3) Age 18 years or older.

The exclusion criterion was unwillingness to participate in the study.

Informed verbal consent was obtained from each participant prior to urine collection. Participants were required to provide their demographic data (gender and age) and a urine sample after completing the physical examination. Data collectors explained the study’s purpose and the consent procedure in the participants’ local language to ensure clear understanding. Verbal consent was documented by participants signing and dating the informed consent forms. Ethical approval for the study and the verbal consent procedure was obtained from the Ethics Review Committee of Beijing University of Traditional Chinese Medicine (approval number: 2020BZYLL0301).

### Urine sample collection

Participants collected midstream morning urine in the hospital toilet using a disposable urine cup. They then transferred a portion of the urine into a standard 10 mL disposable urine collection tube provided by the hospital, with an average transferred volume of 8–10 mL. After collection, participants promptly handed the urine samples to the investigators.

The investigators tested the chemical parameters of the urine within 2 hours of collection and measured Uturb and Ucol within 4 hours.

### Urine chemical analysis

A subset of urine samples was randomly selected for chemical analysis. These samples were tested without any modifications or alterations. Semi-quantitative measurements of Ucol and Uturb, along with 11 urine dry chemical parameters, were obtained by professional testers in the laboratory using a Sysmex UC3500 urine analyzer (Sysmex & Company, Kobe, Japan). The instrument was calibrated with quality control samples before testing.

The Sysmex UC3500 classified Uturb detection into four levels: clear, turbid 1 + , turbid 2 + , and turbid 3 + . Ucol detection was categorized into seven levels: light yellow, straw yellow, yellow, amber, dark brown, red, and other.

### The visual assessment of urine turbidity

**Fig 1:**
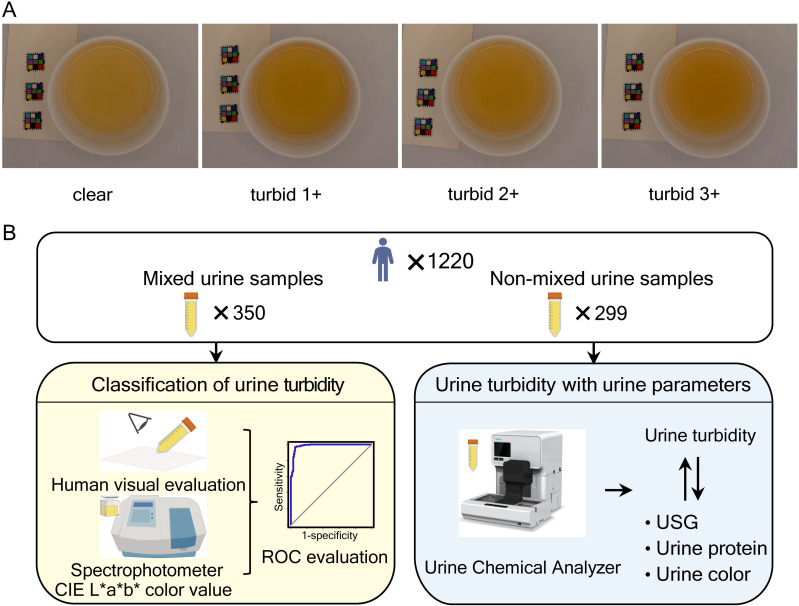
Examples of different Uturb (A) and consort diagram for the study (B).

Urine turbidity was assessed visually by observing the sample through a transparent container. Urine was collected in a 10 mL sharp-bottomed transparent centrifuge tube. A piece of black-printed white paper (Song-style font, size 12) was placed approximately 1 cm behind the pointed end of the centrifuge tube for observation under D65 lighting conditions. Compared to a centrifuge tube containing water, the urine sample was classified as clear if the black text on the paper was legible through the tube. A second investigator independently performed the same procedure without knowing the first investigator’s result. If discrepancies arose between the two assessments, the urine was classified as turbid. Samples classified as turbid were further categorized into three levels: turbid 1 + , turbid 2 + , and turbid 3 + , based on agreement between the two investigators [10,19,20]. Example plots of the different turbidity levels (Uturb) are presented in Fig 1A.

### Spectrophotometric measurement of urine color

The CIE L*a*b* color values of urine samples were measured using a 3nh spectrophotometer YS6060 (Sanenshi Technology Co., Ltd., Shenzhen, China). As the spectrophotometer’s cuvette required a 15 mL sample volume, but individual urine samples were only 8–10 mL, 2–3 urine samples with similar color and turbidity (as assessed visually) were combined and measured together.

The sample combining method was based on the following criteria:

Turbidity matching: Two independent investigators visually assessed the turbidity under D65 lighting using the method described above, classifying it into four levels (0–3).

Color matching: Using the eight-point urine color scale developed by Armstrong et al. [28], two independent investigators matched the urine color to a urine color chart under D65 lighting, categorizing it into eight levels (1–8).

Consistency requirement: The difference in assigned levels between the two investigators must not exceed 1 level.

Mixing process: Urine samples with the same turbidity and color levels were selected and combined. The mixed urine samples were then homogenized using a QT-1 vortex mixer (Qite Analytical Instruments Co., Ltd., Shanghai, China) at 2500 rpm for 30 seconds to ensure uniformity.

### Statistical analysis

Data analysis was conducted using IBM SPSS Statistics 25 (IBM Co., USA). Correlation Analysis: Spearman correlation analysis was performed to assess the relationship between visually detected Uturb and the urine CIE L*a*b* color values measured by the spectrophotometer. Regression Analysis: a standard multiple regression model was developed with the Enter method. The model was constructed to evaluate the strength of the relationship between Uturb as the predictor variable and urine CIE L*a*b* color values as the criterion variable. Receiver Operating Characteristic (ROC) Analysis: The ability of the L* value to assess Uturb was evaluated using an ROC curve, where the L* value served as the predictor variable matched to different Uturb levels from visual examination. The optimal cutoff value for the L* value was determined using the maximum sensitivity and specificity approach. An area under the curve (AUC) of ≥ 0.90 was considered excellent, 0.80–0.89 as good, and 0.70–0.79 as fair [29]. Lastly, spearman correlation analysis was conducted to evaluate the relationship between semi-quantitative Uturb measured by the urine chemical analyzer and urine dry chemical parameters. Additionally, a Mann-Whitney U test was performed to compare urine dry chemical parameters between different Uturb levels. A *p* level of 0.05 was considered statistically significant. The consort diagram for the study is presented in Fig 1B.

## Results

A total of 1,339 individuals from a university in Beijing underwent the physical examination. Of these, 119 cases were excluded, including 96 due to menstruation and 23 due to insufficient urine samples. Ultimately, 1,220 participants (382 males and 838 females, with a mean age of 23.66 ± 2.53 years) were included in the study, providing 1,220 urine samples.

Urine chemical analysis was conducted on 299 randomly selected samples using a urine chemical analyzer. Due to the volume requirements of the spectrophotometer cuvettes, 2–3 urine samples with similar color and turbidity were combined and measured together. This resulted in 350 mixed urine samples, which were subsequently analyzed. The Ucol of the mixed urine samples were measured using a spectrophotometer, and Uturb was assessed visually.

### Relationship between Uturb assessed through visual examination and Ucol measured by spectrophotometry

A total of 350 mixed urine samples were evaluated for Uturb through visual examination, and their CIE L*a*b* color space values were measured using a spectrophotometer. The mean, standard deviation, and two-tailed Spearman correlation coefficients for the CIE L*a*b* values and Uturb data are summarized in Table 1.

**Table 1:** Means, standard deviations, and Spearman’s correlation coefficients for CIE L*a*b* color values and Uturb (n = 350).

Variable	Means	SD	Uturb	L*	a*	b*
Uturb	0.700	1.039	–			
L*	86.128	14.555	-0.842**^a^	–		
a*	1.274	3.916	0.751**	-0.832**	–	
b*	31.802	13.355	0.651**	-0.884**	0.774**	–

^a^**: *p* < 0.01.

Uturb showed a strong negative correlation with the L* value (lightness) and a positive correlation with both the a* value (redness) and the b* value (yellowness).

**Fig 2:**
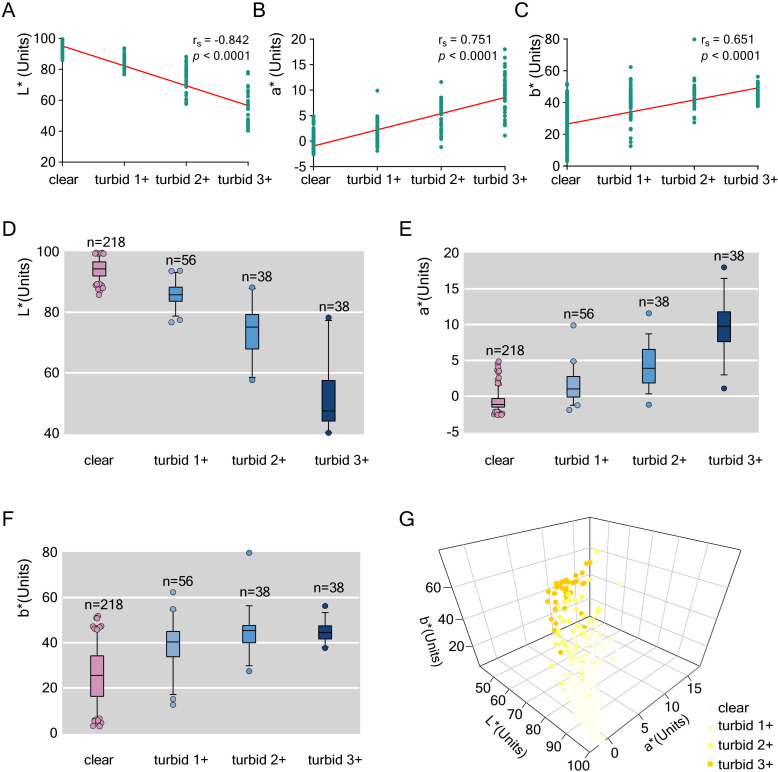
CIE L*a*b* color space values of urine measured by a spectrophotometer showed significant correlations with Uturb as observed through visual examination. (A) The increase in Uturb was significantly correlated with a decrease in urine brightness (L*). (B) The increase in Uturb was significantly correlated with an increase in the red component of urine (a*). (C) The increase in Uturb was significantly correlated with an increase in the yellow component of urine (b*). (D) The boxplot of the decreasing trend of L* values under turbidity gradients. (E) The boxplot of the increasing trend of a* values under turbidity gradients. (F) The boxplot of the increasing trend of b* values under turbidity gradients. (G) \The relationship between Uturb and Ucol in the CIE L*a*b* color space.

As Uturb increased, a clear trend was observed where Ucol became darker, reflected by a linear decrease in the L* value (Fig 2A and Fig 2D). Urine samples with higher turbidity, as assessed visually, exhibited significantly lower L* values, indicating a strong correlation between increased turbidity and reduced brightness.

Additionally, as Uturb increased, the a* value (redness) alsosignificant increased (Fig 2B and Fig 2E). A positive correlation was also observed between Uturb and the b* value (yellowness) (Fig 2C and Fig 2F).

The relationship between Uturb and Ucol was further illustrated in the CIE L*a*b* color space(Fig 2G), showing that urine samples with darker colors were more likely to have higher turbidity levels.

A standard multiple regression model (Enter method) was constructed to evaluate the predictive relationship between the CIE L*a*b* color parameters and Uturb. Standardized beta coefficients were calculated for each parameter. The results indicated that L* (lightness) was a significant predictor of Uturb (standardized coefficient β = -1.030, *p* < 0.0001), as was a* (redness) (standardized coefficient β = -0.129, *p* = 0.035). However, b* (yellowness) was not a significant predictor (standardized coefficient β = 0.013, *p* = 0.675) (Table 2).

**Table 2:** A standard multiple regression analysis results of urine CIE L*a*b* color space value and Uturb (n = 350).

	Non-standardized coefficient β		Standardized coefficient β	*p*-Value	F value of mode	*p*-Value of mode	R^2^	Adjusted R^2^	VIF^a^
Constant	7.053	0.404		0.000	623.270	0.000	0.919	0.844	
L*	-0.074	0.004	-1.030	0.000					8.092
a*	-0.034	0.016	-0.129	0.035					8.266
b*	0.001	0.002	0.013	0.657					1.767

^a^VIF: Variance Inflation Factor.

The regression model was found to be statistically significant in predicting Uturb (F = 623.270, *p* < 0.0001, adjusted R² = 0.844) (Table 2). This result indicates that 84% of the variance in Uturb could be explained by the three predictor variables (L*, a*, and b*). The adjusted R² value, which accounts for sample size and the number of predictors, was used to calculate this percentage of explained variance.

### Accuracy of L* value for assessing Uturb

**Fig 3:**
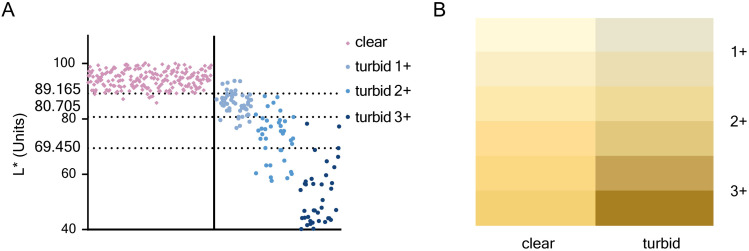
ROC analysis confirmed the role of the L* value in assessing Uturb. (A) Distribution of urine samples with varying turbidity levels based on the L* value. (B) Example images of clear and turbid urine samples with the similar color measured using a spectrophotometer.

The ROC analysis revealed the optimal L* value cutoff for identifying Uturb was 89.165 (AUC = 0.984). L* < 89.165 offered excellent sensitivity and excellent specificity (Table 3, Fig 3A). Additionally, for turbid urine, the optimal L* value cutoff for distinguishing between turbid 2 + and 3 + was 80.705 (AUC = 0.958). L* < 80.705 demonstrated excellent sensitivity and specificity. The optimal L* value cutoff for identifying turbid 3 + was 69.450 (AUC = 0.971), with L* < 69.450 showing excellent sensitivity and good specificity. Simulation examples of clear and turbid urine samples with similar Ucol values (similar b* values) as measured by the spectrophotometer are shown in Fig 3B.

**Table 3:** Urine samples classified according to Uturb through visual examination and urine L* value; followed by metrics from ROC analysis.

	Diagnostic Standard		Metrics from ROC analysis				
	clear	turbid	Sensitivity	Specificity	Accuracy	PPV^a^	NPV^b^
L* ≥ 89.165	211	7	0.968	0.947	0.960	0.968	0.947
L* < 89.165	7	125					
	turbid 1+	turbid 2 + /3+					
L* ≥ 80.705	51	6	0.921	0.911	0.917	0.933	0.895
L* < 80.705	5	70					
	turbid 1 + /2+	turbid 3+					
L* ≥ 69.450	83	2	0.947	0.883	0.902	0.766	0.976
L* < 69.450	11	36					

^a^PPV: Positive predictive value.

^b^NPV: Negative predictive value.

### Relationship between Uturb and urine dry chemical parameters

Urine dry chemical parameters, the semi-quantitative Ucol and Uturb were measured in 299 urine samples using the Sysmex UC3500 urine analyzer. The results of the Spearman correlation analysis (Table 4) showed that Uturb was moderately positively correlated with USG and Ucol. Additionally, Uturb exhibited a positive correlation with protein and a negative correlation with pH.

**Table 4:** Spearman correlation analysis results of Uturb and urine dry chemical parameters (n = 299).

	Urobilinogen	Occult blood	Bilirubin	Ketone body	Glucose	Protein	pH	Nitrite	WBC^c^	USG	Ucol
r_s_^a^	0.023	0.082	0.112	0.074	-0.026	0.242**^b^	-0.201**	0.061	0.028	0.323**	0.397**
*p*	0.690	0.160	0.053	0.201	0.652	0.000	0.000	0.296	0.624	0.000	0.000

^a^r_s_: Spearman’s rho coefficient.

^b^**: *p* < 0.01.

^c^WBC, White blood cell.

**Fig 4:**
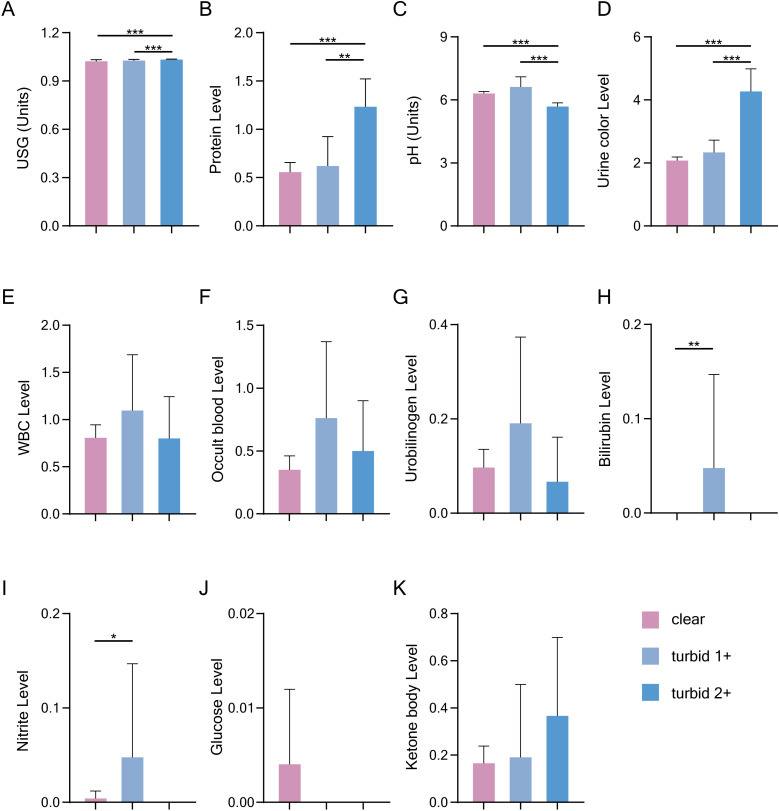
Uturb showed significant correlations with urine dry chemical parameters. The Mann-Whitney U test analysis demonstrated positive correlations of Uturb with USG (A), urine protein (B), and Ucol (D), and a non-linear relationship with the pH value (C). Data are presented as Mean with 95% CI; *: *p* < 0.05; **: *p* < 0.01; ***: *p* < 0.001.

The samples were divided into three groups based on the degree of turbidity (no turbid 3 + was observed in the samples). A Mann-Whitney U test was applied for comparison, and the results are shown in Fig 4. The bilirubin level in the turbid 1 + group (0.048 vs 0.000) was significantly higher than that in the clear group. In the turbid 2 + group, the USG (1.033 vs 1.023), protein (1.233 vs 0.557), and Ucol (4.267 vs 2.073) levels were significantly higher than those in the clear group, while the pH (5.683 vs 6.308) was significantly lower. Similarly, the protein (1.233 vs 0.619), USG (1.233 vs 1.027), and Ucol (4.267 vs 2.333) levels in the turbid 2 + group were significantly higher than those in the turbid 1 + group, while the pH (5.683 vs 6.619) in the turbid 2 + group was significantly lower than that in the turbid 1 + group.

There was a significant relationship between Uturb and pH. The mean pH value in the turbid 1 + group was the highest, while the mean pH value in the turbid 2 + group was the lowest, and the mean pH in the clear group was intermediate. This suggests that the relationship between pH and Uturb was not a simple linear one. In conclusion, Uturb was influenced by USG, protein, pH, and Ucol.

## Discussion

Our study demonstrates that Uturb is closely associated with Ucol CIE L*a*b* values measured by spectrophotometry. Among these parameters, The L* value is an important predictor of Uturb and can effectively classify different levels of turbidity. This novel method for Uturb classification showed high accuracy. These findings suggest that objectively assessing Ucol CIE L*a*b* values by spectrophotometry is a feasible method for determining Uturb. In addition, the occurrence of Uturb may be related to factors such as USG, protein, pH, and Ucol.

In this study, Uturb was assessed using two methods. For exploring the relationship between Uturb and Ucol, Uturb was evaluated through human visual inspection. Spearman correlations revealed a significant relationship between Uturb and each CIE L*a*b* color parameter, with the strongest correlation observed between Uturb and L* (Table 1). The L* value represents lightness, ranging from white (100) to black (0). During dehydration and other physiological changes, increased urine pigment and solute concentrations result in darker Ucol and decreased L* value. This physiological response aligns with the observed negative correlation between Uturb and L* values. Significant positive correlations were also found between Uturb and both a* and b* values. Darker urine corresponded with increased a* and b* values, indicating a higher likelihood of turbidity.

The correlation analysis findings prompted the development of a standard multiple regression model, which determined that 84% of the variance in Uturb could be explained by CIE L*a*b* color predictors (Table 2). The L* value was identified as the most important predictor of Uturb.

In previous studies, Ucol was measured using spectrophotometry. Zhang N et al. analyzed 413 urine samples from 68 participants aged 18–25 years, with an average L* value of 97.0 [30]. Belasco R et al. examined 151 urine samples from 28 participants aged 20–67 years, reporting an average L* value of 95.8 [31]. Neither study included Uturb measurement. In this study, the average L* value of urine samples in the clear group was 94.3, similar to previously reported values. However, the average L* values in turbid 1 + , 2 + , and 3 + groups were 85.8, 73.4, and 52.3, respectively(Fig 2D). The significantly decreased L* values in the turbid groups confirm that L* is a reliable parameter for predicting turbidity.

In addition, the L* value demonstrated excellent accuracy for Uturb assessment (AUC = 0.960). Previous studies have shown that spectrophotometry is an objective and feasible method for measuring Uturb [25–27]. The spectrophotometer used in this study also operated based on the principle of spectrophotometry. Our findings reinforce the strong correlation between these two urinalysis methods.

In the analysis of the relationship between Uturb and urine dry chemical parameters, Uturb was measured using a urine analyzer and categorized into four levels based on absorbance. Results showed that Uturb may be affected by factors such as USG, protein, pH and Ucol. Higher USG reflects increased concentration of solutes and ions in urine [20], contributing to turbid. The correlation between Uturb and Ucol further supports a traditional Chinese medicine theory darker Ucol correlates with increased turbidity. Modern analysis corroborates this finding, as Ucol reflects solute concentrations [32]. Elevated protein levels were also associated with Uturb. Hahn RG et al. linked proteinuria to USG, osmotic pressure, Ucol, and creatinine [33]. Proteinuria often indicates inflammation or renal dysfunction [34].

The relationship between Uturb and pH was nonlinear. Under physiological conditions, urate crystals contribute to turbidity in acidic urine [35], while phosphate or carbonate crystals are associated with alkaline urine [36,37]. Deitel M et al. found that uric acid crystal precipitation caused pink urine in morbidly obese patients, triggered by decreased pH and increased urine concentration [38]. This finding is consistent with the role of pH in influencing Uturb [39]. It is important to note that Uturb often indicates the presence of diffusely dispersed white blood cells (WBCs) associated with infection. However, the effect of WBC on Uturb was minimal in this study, showing only a slight trend, and was relatively high in the turbid 1 + group. The possible reason was that the number of participants with positive WBC results was relatively small, and the classification of WBC was semi-quantitative.

There are several limitations that should be mentioned. First, a diverse population and individuals with urinary tract infections were not specifically included. Second, for Ucol and Uturb analysis, samples with similar color and turbidity, as visually assessed, were pooled due to the spectrophotometer’s cuvette volume. Third, there was no standardized method available for measuring Uturb, so two methods were used: urine analyzer measurement and human visual evaluation. Finally, as this cross-sectional study was conducted during participants’ daily activities without restrictions, external factors may have influenced the measurement of Uturb.

## Conclusions

Overall, this study demonstrates that objective color assessment using spectrophotometry is a reliable and feasible method for determining Uturb, effectively evaluating two objective physical characteristics of urine simultaneously. Classification criteria for Uturb were established, and the relationship between Uturb and urine dry chemical parameters was elucidated. This research represents a key step toward incorporating Ucol and Uturb into hospital urine reporting systems, while also enhancing public awareness and understanding of the clinical significance of Uturb.

## Supporting information

S1 Data This is the data table used for this analysis. (XLSX)
